# Method of Retaining Teeth after Regulating

**Published:** 1907-06-15

**Authors:** W. S. Locke


### METHOD OF RETAINING TEETH AFTER REGULATING.

BY W. S. LOCKE, D. D. S.

Numerous articles have been written upon the subject of regulating teeth, and the correction of deformities of the mouth, but very little has been said upon the important subject of the retention after regulating, or points most necessary to be observed that the teeth may stand in the desired positions without retention by mechanical appliances.

The art of regulating teeth is nothing as compared with the art of retaining teeth. By mechanical appliances and a few brains it is possible to correct almost any of the deformities of the mouth, but without mechanical appliances, how are you going to make them stay there? Those of you who have done any amount of regulating, no doubt know how difficult it is to arrange the teeth in a manner, and with such an occlusion that they will remain where you put them after the retaining appliances have been removed. You are also familiar with the objections and dangers to these long-standing, mechanical-retaining appliances. When worn for months, as is often necessary, we are likely to find decay underlying bands where cement has dissolved. The tooth brush about such bands does not do its duty, and when these retainers are removed, you will find more or less spacing, to say nothing of the unsightly appearance. As these conditions have presented themselves time and again after teeth have been held in position for but a short while, it has become evident that such methods of retaining are very unreliable.

About five years ago, the writer took up the study of this subject to learn, if possible, more about the physical and pathological changes that take place while moving teeth, and the cause of their return after releasing them, with the view of over- coming it by some treatment or operation.

It is well known that when great enough pressure is placed upon a tooth, the tissue in front of it is broken down and carried away by cells known as osteoclasts, and rebuilt (in the space left by the tooth) by those known as osteoblasts. Upon careful examination the writer first found that before any resorption took place the tissue was greatly ly compressed, even the bony substance (process) showed a great amount amount of elasticity. For example in the separating of teeth for filling the rubber plug of gutta-percha being a light and limited pressure, though it may remain for several weeks, does not cause resorption The septum of bone between the teeth only springs, and when the plug is removed, will push the tooth back to its original position. When greater pressure is placed upon this tissue than it can stand, then an inflamation is set up, and resorption takes place nearest the tooth, while that tissue beyond is greatly compressed. If this pressure is released at any time, this compressed tissue recovers itself and pushes backwards, or returns to its original position, less that amount destroyed by resorption. This shows one reason why teeth move back. Again, when a tooth is moved but a short distance, and not beyond the circumference of its socket, it is hard to retain, because the sides of the socket, or process, act as a spring, forces it to the center of the socket, its former position. On the other side of the tooth another condition presents itself, which you will readily see is a cause for this backward movement. The osteoblasts do not replace the tissue nearly so rapidly as it is carried away on the opposite side, so that in the moving of a tooth space is left behind it, with the exception of the soft tissues, which are dragged along, completely covering the cavity underneath. This condition is more marked when a tooth, or teeth are moved outward. The same condition is presented when the teeth are partially drawn from their sockets, as in drawing a cuspid down into position. The plan I have adopted for overcoming these conditions is as follows:

After the tooth or teeth have been regulated, I adjust retaining appliance, then place the patient under an anesthetic, and with a lance, open the tissues at least one-half the distance the length of the tooth on the lingual side, this opening to be the full width of the root. If the tooth has been moved outward, you will find the lance will readily pass into the position formerly occupied by the tooth.

If the tooth is moved inward after going through the soft tissue, it will be necessary to use a bur. Care should be taken not to touch the peridental membrane, or drill too deep. It is also well to cut through the tissue on the labiar side, especially if the tooth is moved outward, by cutting into the septum on both sides.

After the opening is properly made, it is thoroughly packed with antiseptic gauze or cotton, and re-packed several times until the cavity is completely healed from the bottom. The retaining appliance is then removed, and can be done with perfect safety, providing the force of mastication is in the proper direction and not towards that position they formerly occupied. In drawing a tooth from its socket, I drill into the process near the end of the tooth on both sides, care being taken not to go over the end, to avoid destroying the nerve, or up to the peridental membrane. By doing this, new and firmer granulations are formed, and the tooth will remain in the desired position without any retention.

In order to accomplish the above results, however, there are several important points that must be observed. The occlusion of the teeth must be so arranged that the direction of force will be towards the desired position, and not against it. The eruption of the third molar, or wisdom tooth, should not be allowed to force the bite forward, as it sometimes does, and destroy all that has been accomplished, and that brings to mind that the teeth cannot be placed in an over-crowded condition and be expected to remain there, unless held through life by mechanical retaining appliances

Speaking of this subject, a very prominent orthodontist once said to me, "It is not necessary to hold such t[illegible] retaining appliances forever; only until death is sufficient." Except in cases where deformities are very great, our patients would rather we let their mouths alone than to harness them up with these unsightly appliances, to be worn indefinitely; consequently the success of the orthodontist depends upon his diagnosis and treatment, and if he be wedded to any one method or any one system, he is a failure.

When mechanical retaining appliances are necessary, and they are necessary in almost every case for a short period of time to support the tooth or teeth firmly in position until the new tissues have formed about them, the writer advises only such appliances as are easily adjusted, changed or removed. Gold bands or any cemented appliance can not fill these requirements, and are objectionable for reasons given in the fore part of this paper, namely: decay, spacing, etc.; consequently should be used only when necessary. I have found that gold wire woven about the teeth fills the requirements in most every case. It needs no cementing, will cause no decay, no spacing, no irritation, (if adjusted properly), can be tightened at any time while in the mouth, and holds the teeth firmly and closely together. If desired, a gold wire about 20 gauge can be adjusted about the lingual side of each tooth, that portion passing between the teeth filed flat on the outer surface, leaving the rounded surface next to the tooth, these ends just passing the greatest diameter of the tooth. After all are adjusted, take impression, and after placing the wire bands into it, make model and solder the wires together on lingual side, with only sufficient solder to catch them. Place the appliance in position in the mouth, and with long, thin beak pliers, catch the ends of the wires from the labial side, pressing them firmly to the tooth. If at any time it should become loosened, with the pliers, tighten as above described.

Both of these methods were presented to our local society in Cincinnati some years ago by Dr. M. H. Fletcher, and we should all be greatly indebted to him for them, for when once used and used properly, they will never be discarded. As the ideas were new to me at that time, they have been called in my office, the Fletcher method of retention.

The method of retention by operation which I have described, was not presented to me entirely through investigation, but partially through accident. I had been working upon the idea that the crowded tissues should be cut away, but I did not realize that the space left by the tooth would not be at least partially filled by new tissue within a very short time, but it is a fact that in many cases the entire socket of the tooth underlying the outer tissue, remains for a long period of time apparently unrepaired, and I have found time and again the sockets (especially of the anterior teeth) void of any granular tissue whatever. The case to which I have just referred was that of a superior right cuspid in lingual occlusion, with the other teeth in their proper positions, and with plenty of room for this cuspid. The temporary lateral being in position, I extracted and found but very little root to it, although the patient was twenty-five years of age. I started to draw the cuspid into position, but there being such a dense body of process between the teeth, I decided to cut it out, so that the tooth would come through quickly. I did so, and within a short time the tooth was in position. I allowed it to remain in that position for a few days so that it would not be so sore when placing the retaining appliances. When the patient returned, I noticed a little eruption on the lingual side, just under the outer tissue, and opened it, and to my astonishment, I found a cavity large enough to pass a small lead pencil into, nearly half the length of the tooth, and entirely devoid of tissue. This cavity was perfectly healthy, not a pus sack, as you might think. I curetted it, and packed with antiseptic gauze, and re-packed until it was entirely healed. The patient has never worn a retaining appliance, and there is no tendency for the tooth to return to its former position.

I shall speak of another case where a cuspid erupted outside of the arch, with the lateral in lingual occlusion. Some years before some one had extracted the first bicuspid, with the idea that they would straighten themselves, but instead the back teeth moved forward partially filling the space In regulating, it became necessary to crowd them into position, which made them more difficult to retain. After regulating, I anesthetized the patient, and opened under the cuspid and lateral, also opened through the process on the labial side of both teeth, and treated as I have before described. The retaining appliance was removed in about one month, and notwithstanding the crowded condition of the arch, the teeth have remained in place. Both of these cases which I have just cited were operated upon in April, 1900. The following winter I read a paper upon the subject before the Odontological Society of Cincinnati, and while the subject was thoroughly discussed, I gathered from the remarks that in the opinion of most of the discussers, the operation would be of value only in a small per cent. of cases. I was not discouraged, however, and since that time I have performed the operation upon most all types of irregularities, and many of them a great number of times, becoming more and more confident of success, and while all of the little details have not been worked out, I am satisfied this will be the future, and most reliable method of retaining teeth after regulating.

### DISCUSSION.

Dr. V. Barnes: The method advocated of cutting or lancing after regulating, is not the best practice, as it destroys tissues needed to rebuild attachments. This lancing is recommended only after teeth *are in their normal positions.* Then why is it necessary? If a tooth is moved *through the bone,*

why not expect to find a hollow where the tooth was? Why open into an uninfected space? If those teeth are put into their right places, they will not be so very difficult to retain, and no lancing is required. The essayist has mentioned two retainers, but has shown neither, and from the description given, I must conclude that they are not efficient, particularly in holding rotated teeth, and maintaining the correct relation of teeth and jaws. Personally, I find no one or two retainers sufficient. I use fixed and removable ones, depending on the case, the patient and what has been encountered in the regulating. Have used plates with metal clasps projecting between the teeth and others over the interproximal spaces, clasping the teeth on the buccal surfaces. I have found skeleton retainers, made after the Jackson method, of great service in some instances. But at the root of all retention, we must know that early regulation, *at or before the eruption of the lower incisors*, provides the best retainer, *the first deposit of alveolar bone about the teeth in their correct positions.*

Irregularity is shown in the *temporary teeth*, in the incorrect relations of jaws, and in the *absence of proper growth spaces* that provide room for the regular eruption of the permanent teeth. The time for regulation is as *early as you can handle the little patient.* This should be from four years up to seven; beyond this the highest success should not be expected.

Dr. LOCKE: There are several points, however, I must not allow to go unanswered. He refers to the fact that our patients should be taken in charge at a very early age. I want to say as to that, that a large per cent. of patients do not present themselves for treatment until pride forces them, and then they are in their teens; consequently we find at times conditions where irregularities can not be corrected without extraction to get the best permanent results. He says I place my patients under a general anesthetic. I did not mention when all of the molars are in position, no matter how much they have drifted forward, they cannot be pushed backwards. The moving backwards of a second molar in the average-sized mouth, one-eighth of an inch, could open the bite at the incisors more than one fourth-of an inch.

The curetting of the tissues is to stimulate their new and rapid growth, causing an almost immediate tightening about the teeth.

